# *Wuchereria bancrofti* Lymphatic Filariasis, Barrancabermeja, Colombia, 2023

**DOI:** 10.3201/eid3007.231363

**Published:** 2024-07

**Authors:** José A. Suárez, Jose A. Vargas-Soler, Laura Isabel Manosalva-Arciniegas, Stephanie Becerra-González, Angie L. Ramirez, Tatiana Cáceres, Nicolas Luna, Juan David Ramírez, Alberto Paniz-Mondolfi

**Affiliations:** Universidad Internacional Sek Quito, Quito, Ecuador (J.A. Suárez);; Sistema Nacional de Investigación, Senacyt, Panama (J.A. Suárez);; Universidad de Panamá, Panama City, Panama (J.A. Suárez);; Universidad Industrial de Santander, Bucaramanga, Colombia (J.A. Vargas-Soler);; Fundación Cardiovascular de Colombia, Bucaramanga (J.A. Vargas-Soler, L.I. Manosalva-Arciniegas, S. Becerra-González);; Universidad del Rosario, Bogotá, Colombia (A.L. Ramirez, T. Cáceres, N. Luna, J.D. Ramirez);; Icahn School of Medicine at Mount Sinai, New York, NY, USA (J.D. Ramirez, A. Paniz-Mondolfi).

**Keywords:** *Wuchereria bancrofti*, Colombia, lymphatic filariasis, parasites, nematodes, mosquito-borne, vector-borne infections

## Abstract

We describe a recent case of lymphatic filariasis in Colombia caused by *Wuchereria bancrofti* nematodes. Our study combines clinical-epidemiologic findings with phylogenetic data. Resurgence of lymphatic filariasis may be linked to increasing urbanization trends and migration from previously endemic regions. Fieldwork can be a beneficial tool for screening and containing transmission.

*“…could not avoid a spasm of horror at the sight of men with ruptures sitting in their doorways on hot afternoons, fanning their enormous testicle as if it were a child sleeping between their legs […] well-carried rupture was, more than anything else, a display of masculine honor.”*

*“For a long time and with great pride, the scrotal hernia that many men in the city endured was attributed to the water from the cisterns, not only without shame but even with a certain patriotic insolence.”*

—Gabriel García Márquez, Love in the Time of Cholera

Lymphatic filariasis (LF) is caused by an infection with the mosquitoborne filarial nematodes *Wuchereria bancrofti*, *Brugia malayi*, and *Brugia timori* (*1*). Transmission has occurred in various regions, including Africa, Southeast Asia, and the Pacific basin (*1*). In addition, cases have been documented in specific areas across the Middle East, Caribbean, and South America (*1*,*2*). Historically, LF was endemic in 24 countries within the Americas (*1*,*3*); currently, 4 countries remain endemic for LF (Brazil, Haiti, Guyana, and the Dominican Republic), and 13.4 million persons are at risk for infection (*2*). Aside from Guyana, little is known about the occurrence and endemicity of LF caused by the *W. bancrofti* nematodes in northern South America, particularly in Colombia and Venezuela, where no recent cases were reported. We describe an LF case in Colombia caused by *W. bancrofti* infection.

## The Study

A 14-year-old boy residing in an urban area of Barrancabermeja, Santander, Colombia, located to the east bank of the Magdalena River, sought care for a history of progressive lymphedema lasting for 3 years. His symptoms began after a hunting trip to the San Rafael plateau, a forest area situated in the foothills of the eastern mountain range, ≈37 km west of Barrancabermeja. He was referred to Fundación Cardiovascular de Colombia because of progressive enlargement of both testicles, which had become painful over the previous few weeks, and recurrent episodes of fever, erythema, urticarial-like lesions, itching, and pain in the left lower limb. The patient had previously been under outpatient care with a vascular surgery team, who had considered a diagnosis of arteriovenous malformation. Of note, the patient did not experience symptom onset until age 11.

**Figure 1:**
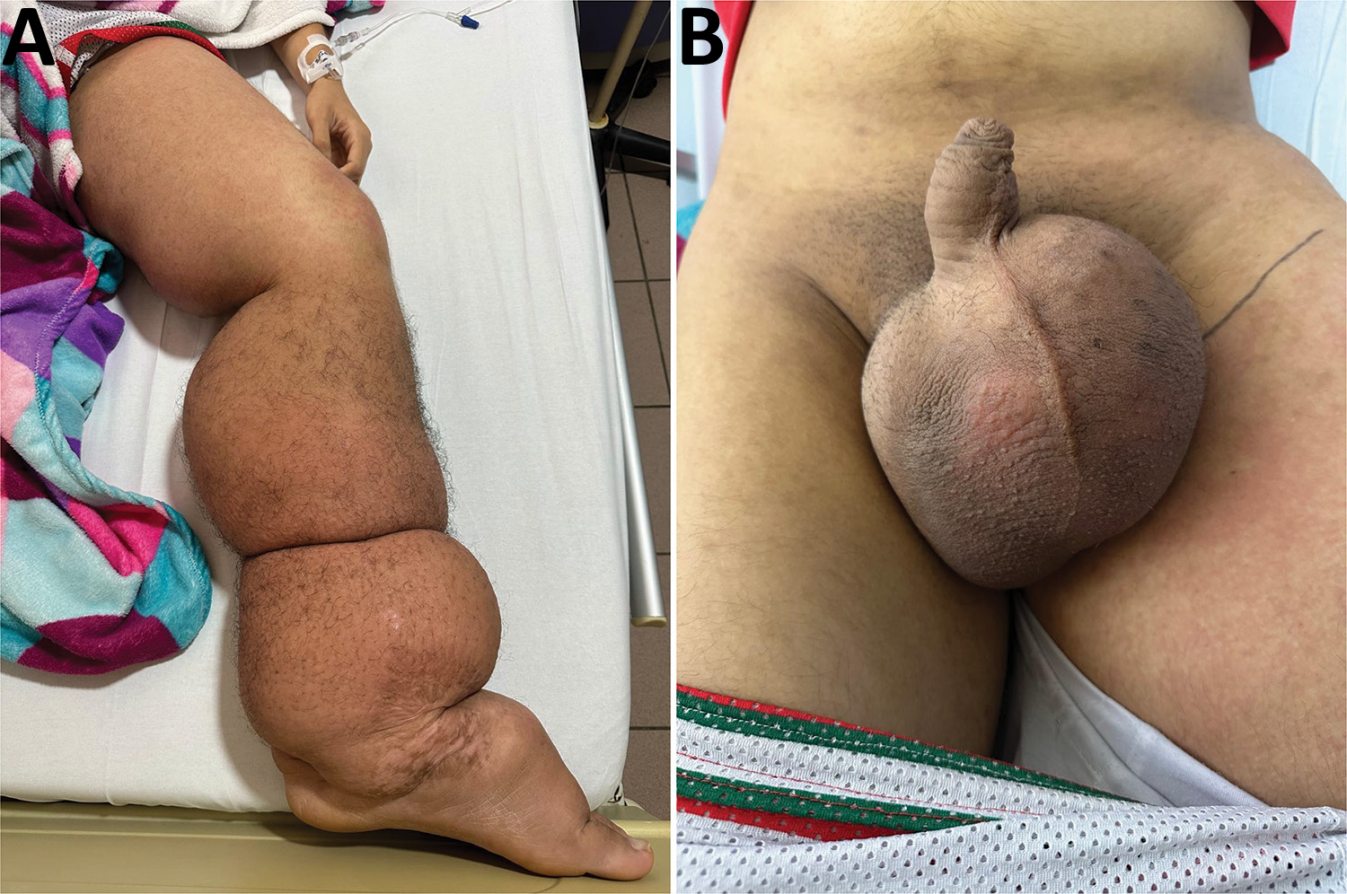
Images of a 14-year-old patient from Colombia experiencing severe progressive lymphedema caused by a *Wuchereria bancrofti* nematode infection. A) Patient’s left lower limb, showing generalized lymphedema extending from the foot to the genital region along with erythematous areas involving the left thigh and areas of perimaleolar hypocromia and hypercromia reflective of post-inflammatory trophic skin changes. B) Severe scrotal edema (hydrocele).

Physical examination of the patient showed extensive lymphedema of the left lower limb, extending from the foot to the genital region, along with areas of induration in the left thigh and painful bilateral inguinal lymphadenopathy (Figure 1, panel A). We also found severe scrotal edema (Figure 1, panel B). Complete blood count results showed leukocytosis with 87.4% neutrophils, indicating neutrophilia (reference range 45%­–70%). All other laboratory test results were unremarkable, including no eosinophilia. Doppler ultrasound revealed bilateral giant scrotal hydrocele. A computed tomography scan of the abdomen and pelvis, performed 3 months earlier, had demonstrated bilateral hydrocele along with left para-aortic, right iliac, and bilateral inguinal lymphadenopathy.

Our suspicion of chronic filariasis prompted nocturnal direct examination by using the Knott concentration method, which yielded inconclusive results (Appendix). We then pursued molecular characterization. We extracted DNA from the patient’s blood sample and conducted a PCR-based filarial detection by using specific primers for *W. bancrofti* nematodes. Those primers targeted the cytochrome c oxidase subunit I, along with short and long fragments of the 18S gene (Appendix).

**Figure 2:**
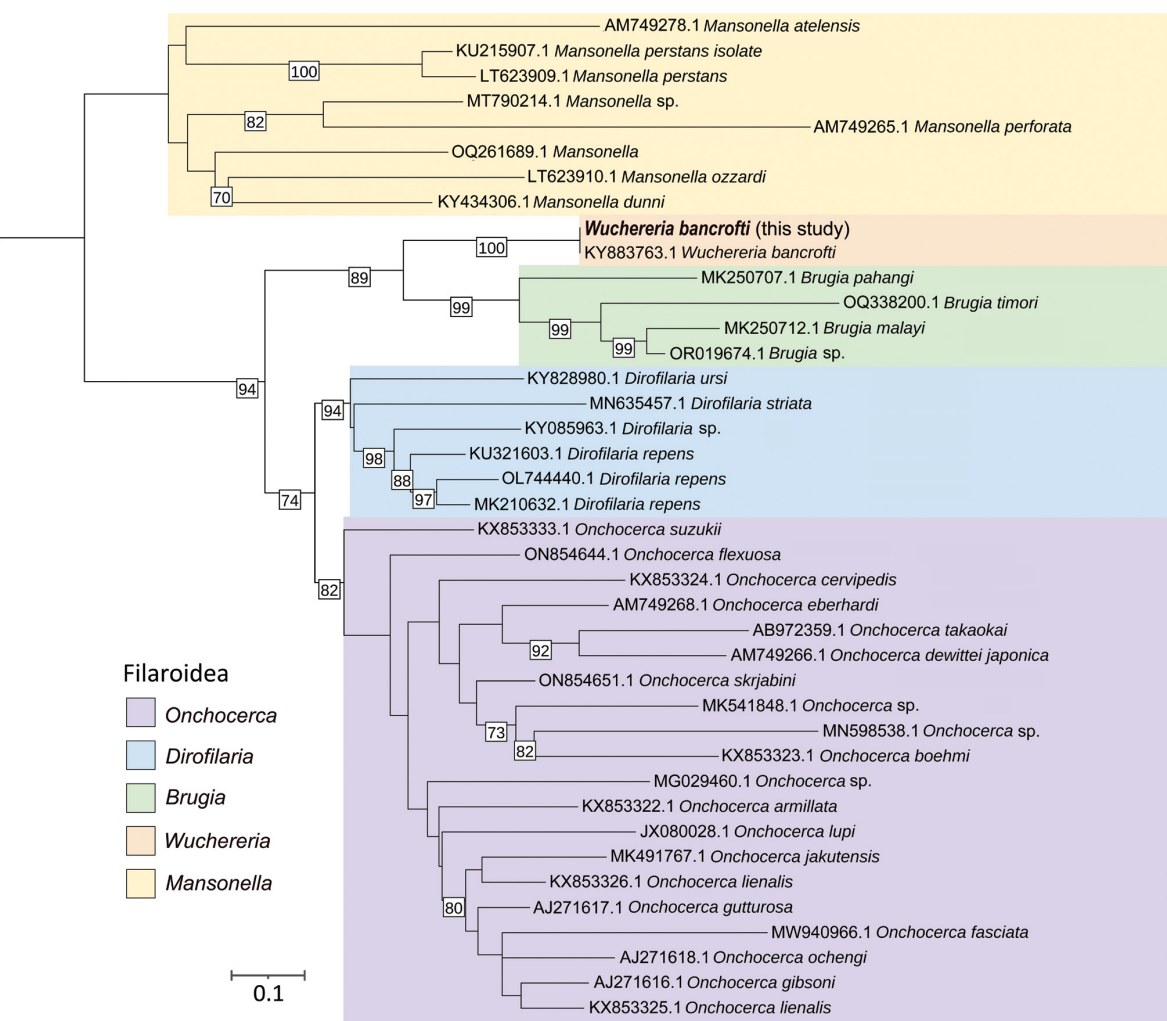
Phylogenetic reconstruction of consensus sequences of filaria, generated from a sample collected from a 14-year-old patient in Colombia (bold text). Cytochrome c oxidase I (COI) gene marker was used for this reconstruction. GenBank accession numbers are provided for reference sequences. Numbers along branch lengths indicate measures of support. Scale bar indicates the distance scale.

We identified the species by using the MinION Sequencing System (Oxford Nanopore, https://nanoporetech.com) for the 3 gene fragments, as described previously (*4*,*5*). We evaluated phylogenetic relationships of the sequenced isolate by comparison against other filarial species, confirming its taxonomic assignment as *W. bancrofti* (Figure 2; Appendix Figure 1). We started the patient on treatment consisting of clindamycin (600 mg intravenously every 8 h for 7 d) and single doses of ivermectin (100 μg/kg) and albendazole (400 mg) while awaiting diethylcarbamazine (DEC), which is currently unavailable in Colombia.

LF was a prevalent clinical condition in Colombia from the 16th Century until the mid-20th Century, prominent in the Caribbean city of Cartagena de Indias, where the initial cases were documented (*6*). However, it was not until 1930 that its endemicity was identified in communities residing along the Magdalena River, specifically in the oil city of Barrancabermeja, where the last case was reported in the late 1940s (*7*). The estimated incidence in that area was ≈16 cases/1000 oil-workers annually, making it one of the country’s major endemic regions (*7*). The incidence of the disease markedly declined during the 1960s and 1970s, resulting in only sporadic and subclinical cases, eventually leading to the apparent disappearance of the disease foci for reasons that remain undetermined. Since that time, no new cases were reported in Colombia until 2016, when a case of giant penile and scrotal lymphedema was documented in a 33-year-old patient in the city of Cali, Valle del Cauca (*8*). However, there was no confirmation of parasite presence or verification of a travel history or residency in endemic areas (*8*).

This case shows the potential for reemergence of LF in Colombia and highlights the clinical characteristics associated with LF. The patient was a 14-year-old boy with chronic manifestations of the disease, including lymphedema and hydrocele, typically observed in the adult population (*9*). The case features dermatolymphangioadenitis attacks characteristic of the acute phase (*9*,*10*). Studies of LF in children suggest advanced stages of lymphedema and the presence of hydrocele, although rare, tend to be more prevalent in male children during puberty or older, which aligns with the observations in our case (*10*,*11*).

DEC is the drug of choice for *W. bancrofti*–caused LF, because of its dual action; primary microfilaricidal and partial macrofilaricidal effects typically result in a 50% reduction in adult filaria (*12*). Because DEC is unavailable in Colombia, we made the decision to initiate treatment with ivermectin in combination with albendazole, leveraging the microfilaricidal properties of ivermectin (*10*). Clindamycin therapy was started to mitigate the risk of secondary bacterial infection because of the patient’s acute dermatolymphangioadenitis. While awaiting DEC availability, initiating doxycycline treatment is being considered, because of its potential for controlling the adult parasite forms (*13*).

Because the patient’s medical history did not indicate travel, we believe the resurgence of LF may be associated with increasing urbanization trends, leading to the breakthrough of new ecologic niches and migration from previously known endemic regions such as Venezuela. From an epidemiologic perspective, it is important to highlight that *W. bancrofti* filaria exhibit a nocturnal periodicity of microfilaremia, which coincides with the peak activity hours of its main vector, *Culex quinquefasciatus* mosquitoes (*14*). *C.* *quinquefasciatus* mosquitoes demonstrate anthropodomestic habits, which are determining factors in the focal transmission of *W. bancrofti* infection in the vicinity of human dwellings (*14*). The patient’s place of residence (Barrancabermeja) is an endemic area not only for *Culex* spp. mosquitoes but also for other vectors involved in the urban transmission of *W. bancrofti* filaria, such as *Aedes* spp. mosquitoes.

## Conclusions

As previously reported, exposure to the LF infection within households appears to be a major contributor to childhood LF infection (*10*,*14*). Investigating familial clustering in this case is warranted. Despite renewed control efforts led by the Global Programme for the Elimination of LF, *W. bancrofti* infection foci persist in the region. Vigilance is crucial to prevent the reactivation of former endemic foci or a resurgence of cases in hypoendemic regions, which is potentially the scenario in this case. Parasite and entomologic surveillance should be promptly established to help develop and implement targeted interventions, including mass drug administration, vector control measures, and other strategic approaches. Such measures are essential for preventing the potential emergence of additional cases of LF within this geographic region of Colombia.

Appendix Additional information about *Wuchereria bancrofti* lymphatic filariasis, Barrancabermeja, Colombia, 2023.
