# Supplementary material for: Wuchereria bancrofti Lymphatic Filariasis, Barrancabermeja, Colombia, 2023
Source: Emerg Infect Dis. 2024 Jul;30(7):1398–401. doi: 10.3201/eid3007.231363 (PMC11210633; doi:10.3201/eid3007.231363)
Supplement: Appendix — Additional information about Wuchereria bancrofti lymphatic filariasis, Barrancabermeja, Colombia, 2023. [file 23-1363-Techapp-s1.pdf]

Article DOI: https://doi.org/10.3201/eid3007.231363

EID cannot ensure accessibility for supplementary materials supplied by authors. Readers who have difficulty accessing supplementary content should contact the authors for assistance.

Wuchereria bancrofti Lymphatic Filariasis, Barrancabermeja, Colombia, 2023

Appendix.

Methods

Sample collection

In this study, we obtained a blood sample from a patient suspected of lymphatic filariasis, which was sent for processing from the Cardiovascular Foundation in Bucaramanga, Colombia.

Microscopy analysis

We conducted a thick blood smear as well as a peripheral blood smear to verify the presence of the parasite. Staining was performed using the GIEMSA dye, and the results were observed through an optical microscope.

DNA extraction

DNA extraction from the blood sample was performed using the High Pure PCR Template Preparation Kit (Roche Diagnostics GmbH, Penzberg, Germany), following the manufacturer's instructions. Subsequently, a PCR assay was conducted using primers targeting the small ribosomal subunit gene of 18S rRNA, an employed genetic marker in helminth identification. To enhance amplicon size and ensure better taxonomic characterization, we employed a combination of previously reported primers: Nem_18S_F (5′-CGCGAATRGCTCATTACAACAGC-3′) and 1289-R (5′- ACTAAGAACGGCCATGCACC-3′) with an amplicon size of 1300bp (*1*,*2*).

To confirm the presence of the parasite, an additional set of primers designed for the amplification of the 18S ribosomal RNA gene of *W. bancrofti* microfilariae specifically was used. Conventional PCR was performed for amplification of an ≈1700 bp fragment using primers

G18S4 and 18P (available at http://nema.cap.ed.ac.uk/biodiversidad/sourhope/nemoprimers.html) (*3*). The amplified fragments were visualized by 1.5% agarose gel electrophoresis, and the DNA concentration was quantified using Qubit.

We also carried out amplification of the mitochondrial cytochrome c oxidase I (COI) gene using forward (5-ATRGTTTATCAGTCTTTTTTTTTTTTTTATTATTGG-3) and reverse (5-GCAATYCAAATAGAAGAAGCAAAAGT-3) primers (*4*). These primers were designed for amplification of a fragment of ≈500bp in filarial positive samples. The amplified fragments were visualized by 1.5% agarose gel electrophoresis, and the DNA concentration was quantified using Qubit.

Sequencing and taxonomic assignment

Sequencing libraries were prepared from the obtained amplicons for rRNA18S and COI. Long-read sequencing on the Oxford Nanopore MinION platform was performed using the MinKNOW 23.04.5 application. Raw Fast5 files were base-called and demultiplexed using Guppy. Subsequently, sequences were quality and length filtered, removing potential chimeric and low-quality sequences.

For bioinformatic analyses, we created a custom database with sequences reported in NCBI for the Filaroidea family for the rRNA18S and COI genes. Taxonomic assignment was subsequently executed through Centrifuge, employing the custom databases, considering reads with a minimum length of 1500bp and 500bp for 18S and COI, respectively. The results of taxonomic assignment were visualized using Pavian (https://fbreitwieser.shinyapps.io/pavian/), and the taxonomic assignments were further validated through BLAST (https://blast.ncbi.nlm.nih.gov/Blast.cgi).

Consensus sequences generation from Oxford Nanopore reads

The reads assigned to *Wuchereria* by centrifuge were filtered using Seqtk (https://github.com/lh3/seqtk). Subsequently, a mapping was performed with Minimap2 (version 2.24) using the selected reference sequences of the small ribosomal subunit gene of 18S rRNA (GenBank: OR662183.2), large ribosomal subunit gene of 18S rRNA (GenBank: PP343069) and the cytochrome c oxidase subunit I (COI) gene (GenBank: PP342541) for *Wuchereria bancrofti*.

From the mapped sequences, a Variant Call Format (VCF) file was generated using Samtools (v 1.17). This VCF file was used to identify differences compared to the reference sequences of *Wuchereria bancrofti*. Finally, Bedtools (version 2.25.0) was employed to generate the consensus sequence, which served as the basis for subsequent analyses.

Phylogeny

We conducted a phylogenetic reconstruction to analyze the evolutionary relationships between *Wuchereria* sequences obtained by 18S (long and short fragments) and COI genes and reference sequences from other Filaroidea groups, including *Onchocerca, Dirofilaria, Brugia* and *Mansonella* species. These sequences were obtained from the 18S rRNA small ribosomal subunit gene and the cytochrome c oxidase subunit I (COI) gene, downloaded from NCBI. For 18S rRNA, sequences shorter than 1000 bp were excluded to ensure that the analysis included significant genetic information of the gene. For COI, sequences shorter than 300 bp were excluded considering that an amplified fragment of ≈500 bp was expected to be obtained. To improve computational capacity and to better elucidate phylogenetic relationships, we carried out a removal of duplicate sequences and clustering of sequences with a percentage of identity greater than 97% using Vsearch (https://github.com/torognes/vsearch). Subsequently, we perform multiple sequence alignment using MAFFT v7.407 and used this alignment to construct a maximum likelihood (ML) tree in IQ-TREE multicore v1.6.12, using the best substitution model and other parameters with default values. The phylogenetic tree was graphically represented in Interactive Tree Of Life (iTOL) v5.

Results

Microscopy analysis for the detection of microfilariae in a blood sample

We conducted a microscopic analysis to confirm the presence of the parasite in the patient's sample. Employing both peripheral blood smear and thick blood smear techniques, coupled with Giemsa staining, we examined the blood sample. The microscopic evaluation revealed a sheathed filariform-like structure for which microanatomical landmarks could not be fully resolved rendering diagnosis inconclusive. Therefore, the sample was analyzed by means of molecular detection by PCR.

Sequencing and taxonomic assignment

The reads obtained through long-read sequencing with Oxford Nanopore technology were taxonomically assigned using Centrifuge against a custom database created from 18S rRNA and COI gene sequences of the filaroidea family. The results of the taxonomic assignment corroborate *Wuchereria bancrofti* as the species involved in the case of filariasis reported here.

Phylogeny

We carried out a phylogenetic analysis of the sequences found for the rRNA18S and COI genes of *Wuchereria bancrofti*. The results obtained from the phylogenetic reconstruction of both molecular markers support the phylogenetic closeness of the sequence obtained here with previously reported *Wuchereria* species in a well-supported monophyletic cluster (Boostrap value >80). This is also supported by the fact that this sequence shows divergence from other filarial species responsible for cases of lymphatic filariasis such as *Brugia malayi* and *Brugia pahangi*. These findings support the hypothesis that the identified parasite in this patient corresponds to *Wuchereria bancrofti*.

References

1. Davidov K, Iankelevich-Kounio E, Yakovenko I, Koucherov Y, Rubin-Blum M, Oren M. Identification of plastic-associated species in the Mediterranean Sea using DNA metabarcoding with Nanopore MinION. Sci Rep. 2020;10:17533. PubMed https://doi.org/10.1038/s41598-020-74180-z

2. Knot IE, Zouganelis GD, Weedall GD, Wich SA, Rae R. DNA barcoding of nematodes using the MinION. 2020. [cited 2023 Sep27]. https://www.frontiersin.org/articles/10.3389/fevo.2020.00100/full

3. Bhandari Y, Dabir P, Nandhakumar K, Dayananda KM, Shouche YS, Reddy MVR. Analysis of polymorphism of 18S rRNA gene in *Wuchereria bancrofti* microfilariae. Microbiol Immunol. 2005;49:909–14. PubMed https://doi.org/10.1111/j.1348-0421.2005.tb03682.x

4. Laidoudi Y, Medkour H, Levasseur A, Davoust B, Mediannikov O. New molecular data on filaria and its *Wolbachia* from Red Howler Monkeys (*Alouatta macconnelli*) in French Guiana-a preliminary study. Pathogens. 2020;9:8. PubMed https://doi.org/10.3390/pathogens9080626

5. World Health Organization. Lymphatic filariasis. 2023 [cited 2023 Sep 25]. https://www.who.int/news-room/fact-sheets/detail/lymphatic-filariasis

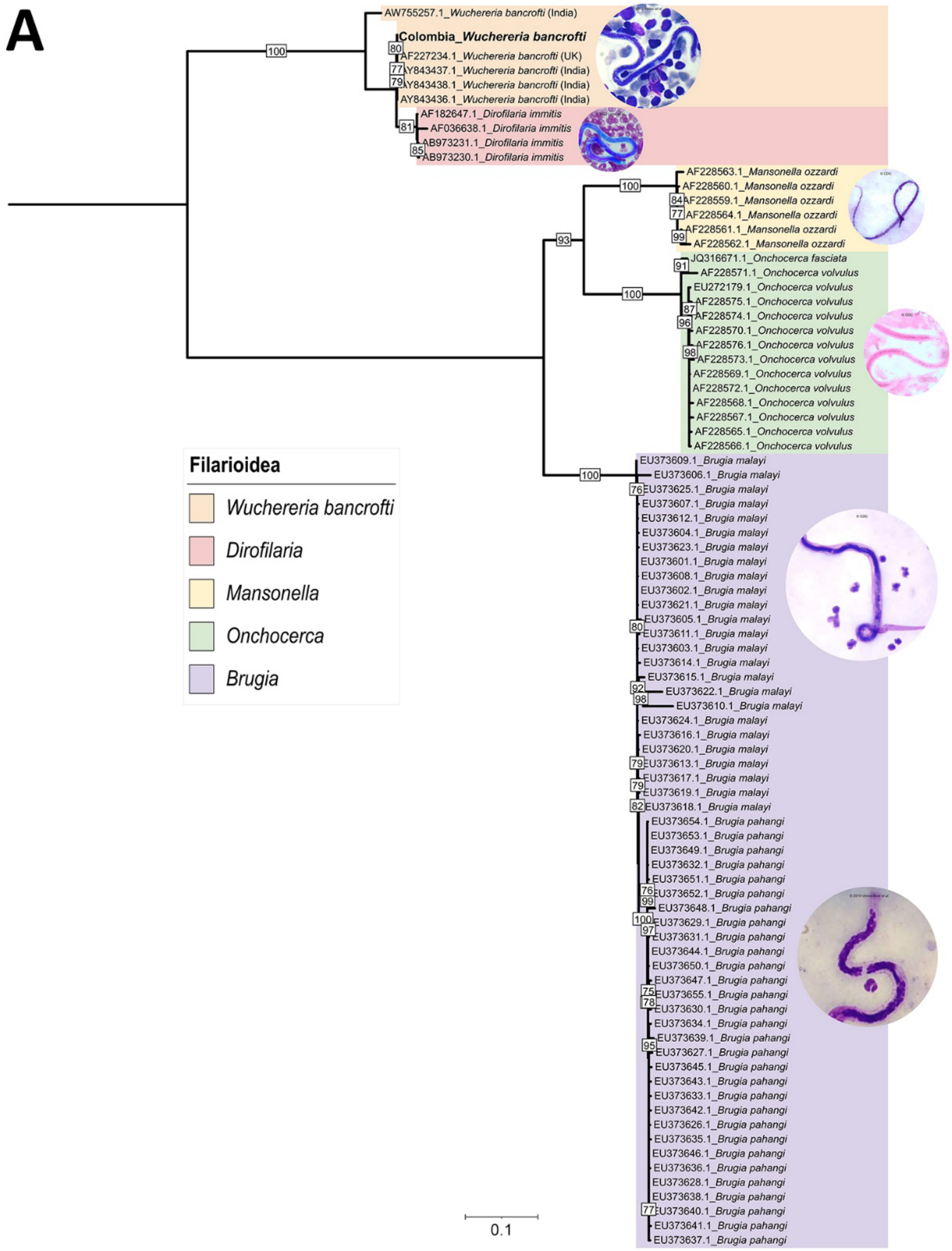

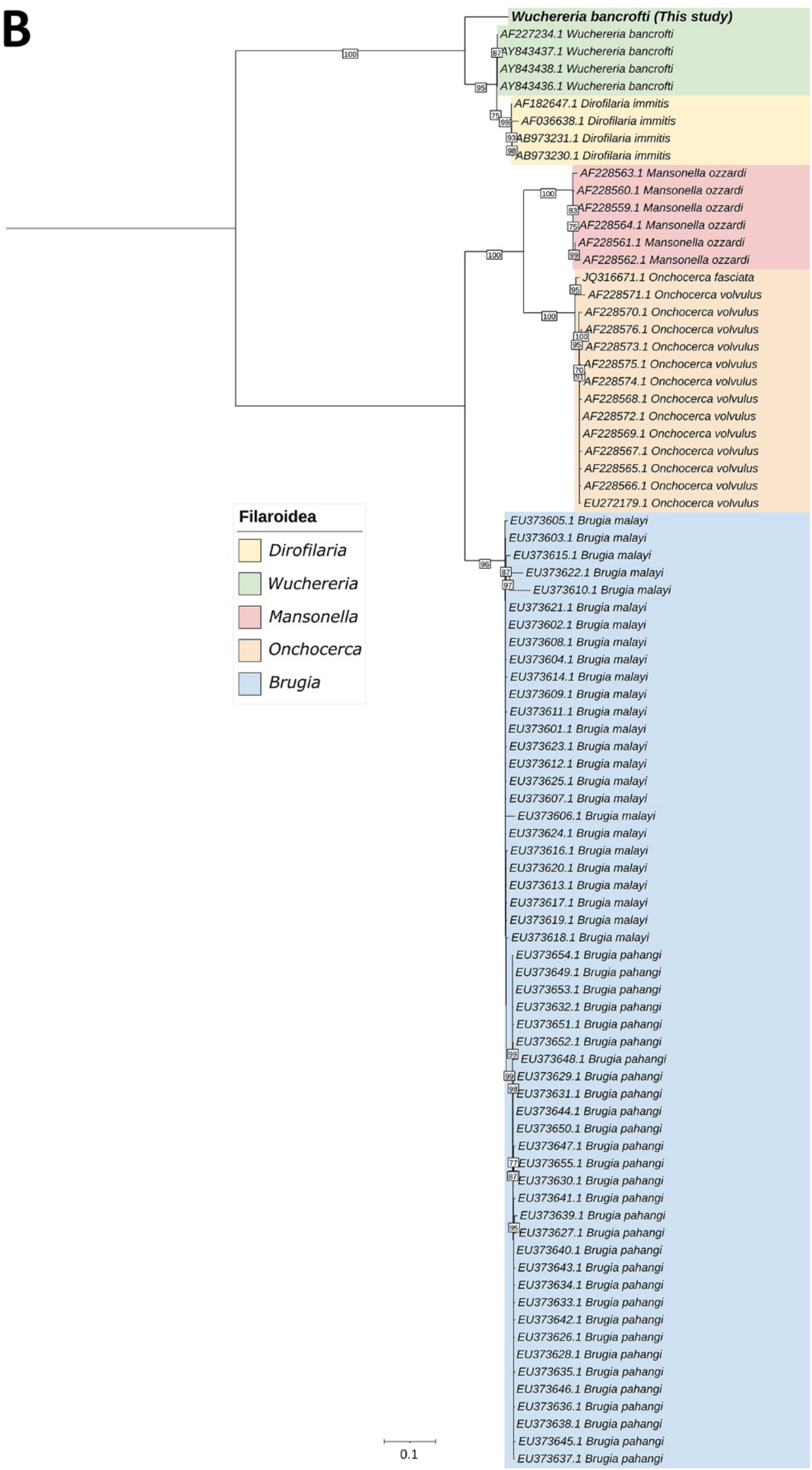

Figure 1. Phylogenetic reconstruction of consensus sequences, generated from a sample collected from a 14-year-old patient in Columbia. 18S Ribosomal RNA Gene used for reconstruction (bootstrap >70) for both panels A) and B).

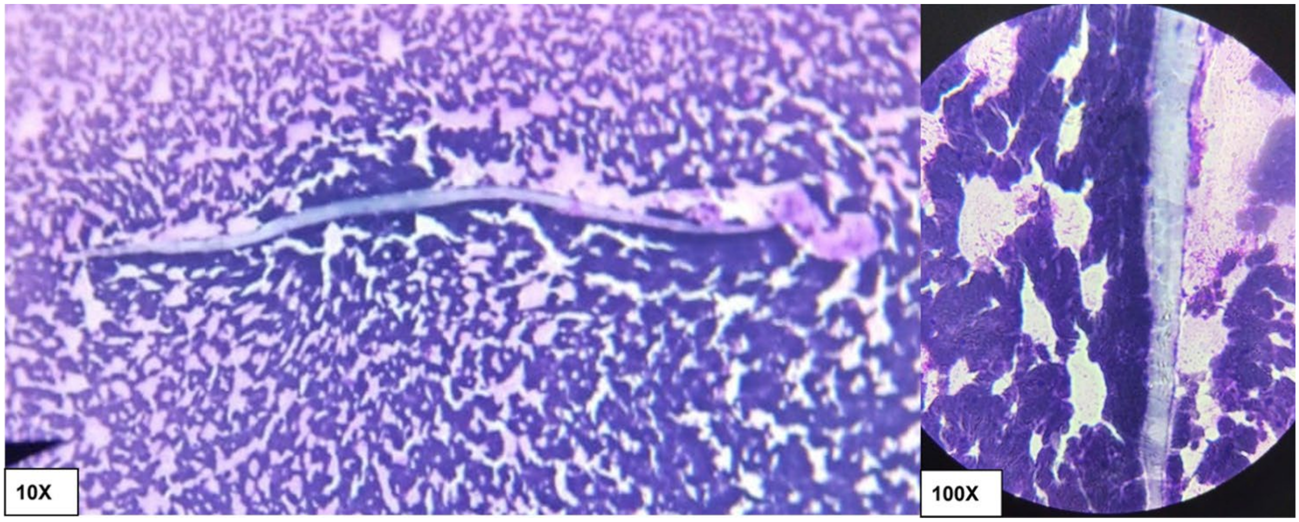

Figure 2. Identification of filariform-like structure in peripheral blood smear. Giemsa 10x and 100x
